# 875. Indirect Treatment Comparison of 48-Week Efficacy and Safety of Cabotegravir + Rilpivirine Long-Acting Every 2 Months to Bictegravir/Emtricitabine/Tenofovir Alafenamide in Suppressed HIV-1 Infected Participants

**DOI:** 10.1093/ofid/ofab466.1070

**Published:** 2021-12-04

**Authors:** Sonya J Snedecor, Melanie Schroeder, Nicolas Van de Velde

**Affiliations:** 1 OPEN Health, Bethesda, MD; 2 ViiV Healthcare, Brentford, UK

## Abstract

**Background:**

Switching to cabotegravir long-acting + rilpivirine long-acting (CAB LA + RPV LA) administered every month (Q1M) has demonstrated non-inferiority in viral suppression versus a range of standard of care (SoC) antiretroviral regimens, including tenofovir alafenamide based regimens, in two pivotal phase 3 clinical trials (ATLAS [NCT02951052] and FLAIR [NCT02938520]). Furthermore, CAB LA + RPV LA every 2 months (Q2M) has demonstrated non-inferiority in maintaining viral suppression compared with CAB LA + RPV LA Q1M in a phase 3b study (ATLAS-2M [NCT03299049]). As bictegravir/emtricitabine/tenofovir alafenamide (BIC/FTC/TAF) was not widely used at study initiation, the regimen was not present in the SoC arms of ATLAS and FLAIR. The objective was to compare efficacy and safety of CAB LA + RPV LA Q2M to BIC/FTC/TAF using indirect treatment comparison.

**Methods:**

Two switch studies appropriate for facilitating indirect comparison to BIC/FTC/TAF were identified via systematic literature review (Molina et al. 2018 [NCT02603120] and Sax et al. 2020 [NCT03110380]). Indirect comparison using a generalisation of Bucher’s methodology to calculate relative risk, odds ratio, and risk differences in efficacy (Week 48 HIV RNA < 50 c/mL and ≥50 c/mL per FDA Snapshot approach and CD4+ cell change from baseline) and safety (discontinuation due to adverse events [AEs] and overall and serious AEs excluding injection site reactions [ISRs]) was conducted. Results for CAB LA + RPV LA Q2M in ATLAS-2M participants with prior integrase inhibitor (INI) exposure, but without prior CAB exposure, were indirectly compared to those with prior INI use in ATLAS and FLAIR via the common CAB LA + RPV LA Q1M comparator and were then indirectly compared to BIC/FTC/TAF via the INI comparator (Figure 1).

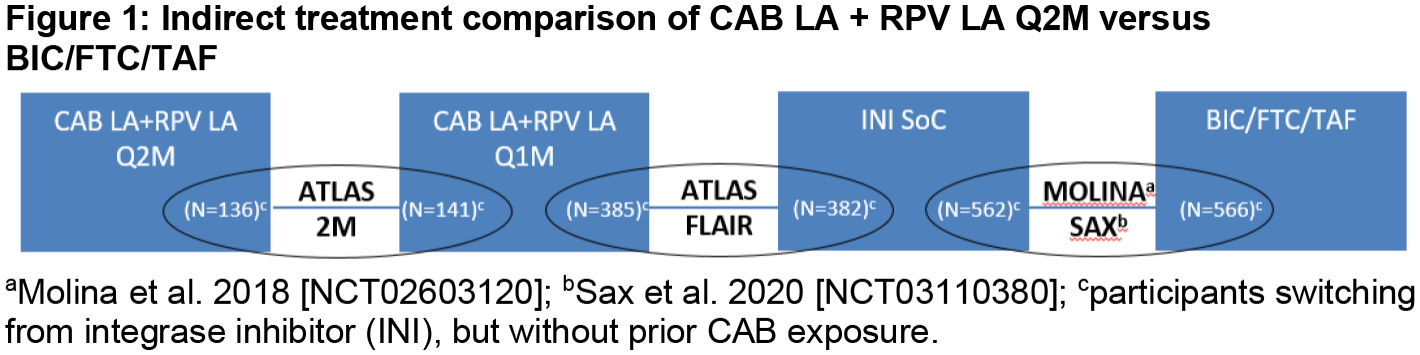

**Results:**

No statistically significant differences in virologic failure, virologic suppression, CD4+ cell change, discontinuations due to AEs, and non-ISR serious/non-serious AEs were found between CAB LA + RPV LA Q2M and BIC/FTC/TAF (Table 1).

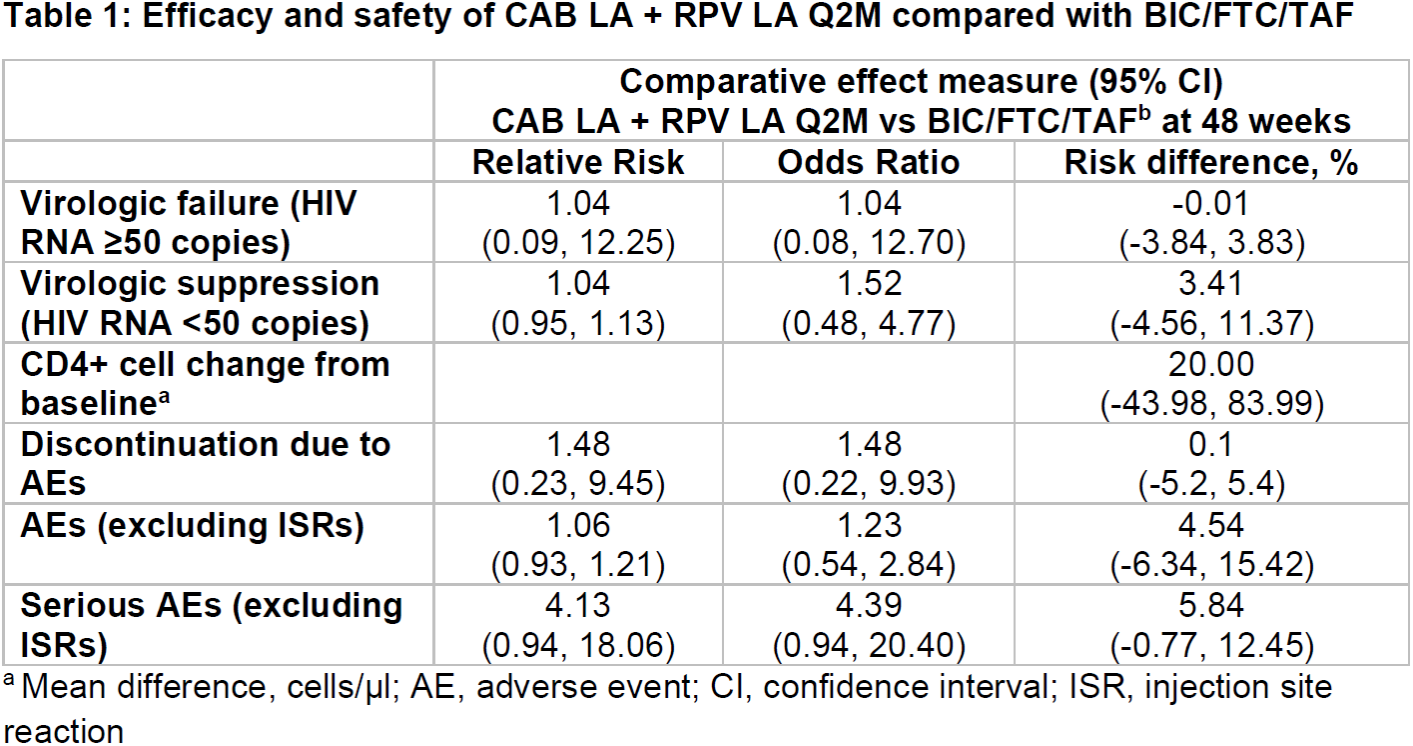

**Conclusion:**

Indirect treatment comparison indicated efficacy and safety of CAB LA + RPV LA Q2M is not different from BIC/FTC/TAF. These regimens will be further compared in a randomized head-to-head non-inferiority trial (SOLAR, NCT04542070).

**Disclosures:**

**Sonya J. Snedecor, PhD**, **ViiV Healthcare** (Other Financial or Material Support, Author’s employer, OPEN Health received funding to execute this study) **Melanie Schroeder, MSc**, **ViiV Healthcare** (Employee) **Nicolas Van de Velde, PhD**, **ViiV Healthcare** (Employee)

